# Environmental and phenotype-related risk factors for owner-reported allergic/atopic skin symptoms and for canine atopic dermatitis verified by veterinarian in a Finnish dog population

**DOI:** 10.1371/journal.pone.0178771

**Published:** 2017-06-01

**Authors:** Johanna Anturaniemi, Liisa Uusitalo, Anna Hielm-Björkman

**Affiliations:** 1 Faculty of Veterinary Medicine, Department of Equine and Small Animal Medicine, University of Helsinki, Helsinki, Finland; 2 Faculty of Agriculture and Forestry, Department of Agricultural Sciences, University of Helsinki, Helsinki, Finland; "INSERM", FRANCE

## Abstract

The aim of this cross-sectional study was to observe whether environmental factors and phenotypic traits are associated with owner-reported skin problems and with veterinary diagnosed canine atopic dermatitis (CAD). Data were collected using the validated online DOGRISK questionnaire. Out of the data that the questionnaire provides for analysis, focus was first turned towards addressing questions regarding ‘Atopy/allergy (skin symptoms)’ using a total of 8643 dogs: 1585 dogs with owner-reported allergic/atopic skin symptoms and 7058 dogs without. A subsequent analysis compared dogs with veterinary-verified CAD (n = 322) as a case group against the 7058 dogs without owner-reported skin symptoms. The association between 21 factors related to the environment, canine phenotypes and breed groups within both populations were analysed using univariable and multivariable logistic regression. The environmental factors that showed a significant inverse association with the risk of owner-reported allergic/atopic skin symptoms were as following: whether the dog was living in a detached house, whether there were other dogs in the household, and whether the dog was born in the current household. Having over 50% white colour in the coat and living in an extremely clean household were significantly associated with an increased risk of owner-reported allergic/atopic skin symptoms. The five breeds demonstrating the highest proportion of owner-reported allergic/atopic skin symptoms were West Highland white terrier, Boxer, English bulldog, Dalmatian and French bulldog. The Fédération Cynologique Internationale dog breed groups 3 (Terriers) and 6 (Scent hounds and related breeds) showed a significantly higher risk for owner-reported allergic/atopic skin symptoms than mixed breed dogs. In the second population, the inverse association was observed between the risk of CAD and the presence of other dogs in the household, and whether the dog had been born in the current household. The results indicate that some environmental factors and canine phenotypes are associated with CAD and owner-reported skin symptoms, but they still do not prove causality.

## Introduction

Canine atopic dermatitis (CAD) affects up to 10% of dogs [1] and it is described as a genetically predisposed inflammatory and pruritic dermatitis with characteristic features related to IgE antibodies usually directed against environmental allergens [2]. Atopic dermatitis (AD) in both humans and dogs is influenced by both genetic and environmental factors as the aetiopathogenesis is very complex [3, 4]. The prevalence of AD in humans has nearly tripled during the past three decades in industrialized countries [5], and the trend seems to be similar in dogs. The incidence of human atopy and asthma is much higher in Western countries and urban areas [6–11] whereas living on a farm or even having regular contact with a farming environment protects humans from atopic diseases [12–15]. Having animals in the household may have a protective effect against the development of allergic diseases in humans, as has been reported regarding both dogs [8, 16–20] and cats [16, 18, 21, 22]. Similar results were also found in a canine study, where the presence of cats and/or other dogs in the household were inversely associated with CAD [23]. A positive history of parental AD and allergies in humans has been found to be associated with the risk of developing AD [17, 20, 24–28], which highlights the role of genetics along with environmental factors.

The clinical signs of CAD are typical but there are differential diagnoses that show similar signs. Both owner-reported and veterinary-diagnosed skin symptoms are often related to allergy, including CAD or adverse food reactions, and are often accompanied with a secondary infection, mainly bacterial folliculitis associated with Staphylococcus pseudintermedius and Malassezia dermatitis [29, 30]. But owner-reported and veterinary-diagnosed skin symptoms can also be caused by e.g. parasites, behavioural or stress induced scratching and licking [31] or even idiopathic seborrhoea or hypothyroidism or [32, 33]. In Finland we do not have canine fleas but dogs are sometimes affected by fleas from wild mammals [34]. *Demodex* mites are a more common reason for dermatitis [35]. Even when a veterinarian makes the diagnosis, it is not always ascertained what was the primary cause of the skin symptoms.

The aim of this study was to find environmental factors and phenotype characteristics associated with canine skin problems reported by the owner and with veterinary diagnosed CAD in a Finnish dog population. The hypothesis was that the risk of allergic/atopic skin symptoms and CAD are associated with environmental factors related to puppyhood, household, and dog care-related conditions, in addition to dog characteristics.

## Materials and methods

This was a cross-sectional epidemiological study based on the validated large scale internet-based DOGRISK questionnaire for dog owners [36], which was released at the Faculty of Veterinary Medicine of University of Helsinki in December 2009. It contains questions about dogs’ diseases, living environment, and nutrition, with a generated excel data sheet of 1332 variables. The questionnaire has been open for all Finnish dog owners. The questionnaire was advertised widely throughout the country and it was not directed toward any special group of owners. Flyers asking dog owners to complete the internet questionnaire were given to owners through a Finnish dog food distribution car, at national and international dog shows, in dog parks, animal clinics, and pet shops. Also, the questionnaire was advertised in different dog magazines, in articles published in dog magazines, and on television. The DOGRISK questionnaire informs the dog owners’ that the results will be published in national and international journals. By filling in the questionnaire they gave their consent to this. A separate statement from the ethics committee was not needed for this kind of study in Finland (Ethical committee decision 29.4.2016). As a part of the DOGRISK questionnaire reliability and validity testing [36], a diagnosis-verifying follow-up questionnaire was sent out to 1551 owners that had answered that their dogs suffered from ‘Atopy/allergy (skin symptoms)’. The owners of 578 dogs answered to the follow-up questionnaire. The verified CAD patients were used as a second population in this study.

Thereby we had two populations in this study. The DOGRISK questionnaire population is referred to as the ‘owner-reported allergic/atopic skin symptoms’ population as the owners here answered positively to a question that was given as ‘Does your dog suffer from atopy/allergy (skin symptoms)’ (first case group, n = 1585). The second population was based on the follow-up question, and included only dogs whose diagnosis of atopy was verified by a veterinarian (veterinary-verified CAD case group, n = 322). The control group for both populations consisted of 7058 dogs with no owner-reported skin symptoms.

All 21 categorical variables used in this study are listed in Supporting information S1 Table. In addition, age was used as a continuous variable. When the answer ‘I do not know/remember’ was available and selected by the owner, it was omitted from statistical analyses. In the dichotomous questions, “Living with other dogs”, “Living with other animals” and “Born in owner family”, which offered “yes” or “no” as options, the missing answer was interpreted as a “no”.

The proportion of owner-reported allergic/atopic skin symptoms were calculated for the top 20 dog breeds. Then, the risk of owner-reported allergic/atopic skin symptoms was compared between dog breed groups according to the Fédération Cynologique Internationale (FCI) [37] and mixed breed dogs using a univariable logistic regression model. The number of cases vary for every analysed question, as answering them was not mandatory. Dogs with missing data were dropped out from their respective analysis.

### Statistical analyses

First, a univariable logistic regression was run individually for each variable in order to determine its association with both the owner-reported skin-symptom status and the veterinary-verified CAD. All risk factors that had a P < 0.2 and less than 1500 missing cases (less than 1500 owners had left the question unanswered) were included into the multivariable logistic regression analysis using the enter method. P < 0.05 was here considered as statistically significant. The quality of the fit of the final model was determined by the following criteria: a smaller P-value in the Omnibus test of model coefficients, a larger value in the Hosmer and Lemeshow test, and the closer the value to 100% in Nagelkerke’s R^2^ [38].

The proportion of owner-reported allergic/atopic skin symptoms in this data was calculated breed-specifically for each breed where there were over 40 answers. After that, all breeds were categorized according to FCI breed groups and the risk of owner-reported allergic/atopic skin symptoms was analysed in every group compared to the mixed-breed dogs using a univariable logistic regression model. P < 0.05 was considered as statistically significant. All analyses were performed using SPSS software (version 22, IBM SPSS Statistics. Chicago, Ill., USA)

## Results

**Fig 1:**
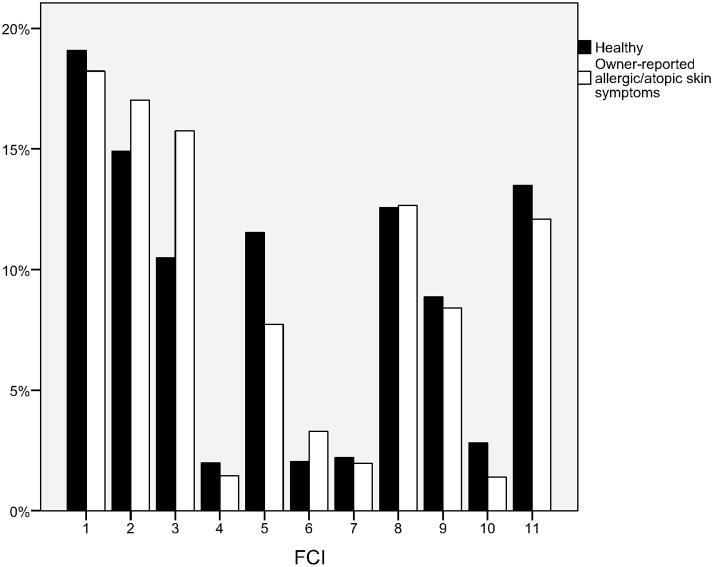
Percentage of atopic/allergic and healthy dogs divided by FCI dog breed groups 1–10 and by a mixed breed group. 1 = Sheepdogs and cattledogs (except Swiss cattledog), 2 = Pinscher and Schnauzer—Molossoid and Swiss Mountain and cattledogs, 3 = Terriers, 4 = Dachshunds, 5 = Spitz and primitive types, 6 = Scent hounds and related breeds, 7 = Pointing dogs, 8 = Retrievers—Flushing dogs—Water dogs, 9 = Companion and toy dogs, 10 = Sighthounds, and 11 = Mixed breeds.

The five breeds that suffered most often from owner-reported allergic/atopic skin symptoms as percentage within the breed were: 1. West Highland white terrier, 2. Boxer, 3. English bulldog, 4. Dalmatian, 5. French bulldog. The 20 breeds with the highest proportion of owner-reported allergic/atopic skin symptoms from the DOGRISK data are shown in Table 1. When compared to mixed breed dogs, the risk of owner-reported allergic/atopic skin symptoms among the FCI breed groups was highest in group 3 (Terriers) and group 6 (Scent hounds and related breeds), and lowest in group 5 (Spitz and primitive types) and group 10 (Sighthounds), compared to mixed breed dogs. Results are shown in Table 2 and Fig 1.

**Table 1:** Within breed percentage of dogs with skin symptoms in the 20 dog breeds with most owner-reported allergic/atopic skin symptoms. Only breeds with more than 40 dogs were included in the table.

	Breed	N	Owner-reported allergic/atopic skin symptoms (% within the breed)
**1.**	West Highland white terrier	67	41.8
**2.**	Boxer	85	40.0
**3.**	English bulldog	44	36.4
**4.**	Dalmatian	55	34.5
**5.**	French bulldog	71	33.8
**6.**	Staffordshire bull terrier	139	32.4
**7.**	Parson Russell terrier	74	32.4
**8.**	German shepherd	520	31.3
**9.**	English springer spaniel	52	30.8
**10.**	American Staffordshire terrier	53	30.2
**11.**	Welsh springer spaniel	45	28.9
**12.**	Great Dane	79	25.3
**13.**	Miniature Pinscher	90	23.3
**14.**	Labrador retriever	293	21.5
**15.**	Jack Russel terrier	121	21.5
**16.**	Newfoundland	53	20.8
**17.**	Lagotto Romagnolo	45	20.0
**18.**	Flat-coated retriever	67	19.4
**19.**	Standard poodle	62	19.4
**20.**	Doberman Pinscher	80	18.8

**Table 2:** Results of univariable logistic regression analysis of owner-reported allergic/atopic skin symptoms in the DOGRISK questionnaire data in FCI breed groups compared to mixed breed dogs (n = 8501).

FCI breed group	Group name	P-value	OR	95% CI
**1**	Sheepdogs and Cattledogs (exp. Swiss Cattledogs)	0.918	**1.01**	0.83–1.23
**2**	Pinscher and Schnauzer—Molossoid and Swiss Mountain and Cattledogs	0.063	**1.21**	0.99–1.49
**3**	Terriers	**<0.001**	**1.64**	1.32–2.03
**4**	Dachshunds	0.368	0.81	0.51–1.29
**5**	Spitz and primitive types	**0.007**	**0.71**	0.56–0.91
**6**	Scent hounds and related breeds	**0.004**	**1.68**	1.18–2.29
**7**	Pointing Dogs	0.732	0.93	0.61–1.41
**8**	Retrievers—Flushing Dogs—Water Dogs	0.437	1.09	0.88–1.35
**9**	Companion and Toy Dogs	0.804	1.03	0.81–1.31
**10**	Sighthounds	**0.007**	**0.53**	0.33–0.84

FCI, Fédération Cynologique Internationale; OR, odds ratio; CI, confidence interval. **Bolded**, P < 0.05.

### Regression analysis of environmental and phenotype related factors

The mean age of dogs with owner-reported allergic/atopic skin symptoms was 4.5 years (SD± 3.05) and dogs without owner-reported allergic/atopic skin symptoms 4.0 years (SD± 3.26). Distributions of all the categorical variables for the larger population of the DOGRISK cases and controls are given in Supporting information S1 Table. The percentage of owner-reported allergic/atopic skin symptoms in the entire dataset was 18.3%. In the univariable analyses, 14 of the 21 variables showed a statistically significant association with owner-reported allergic/atopic skin symptoms (Table 3). All variables were tested for bivariate correlation with each other. All the observed correlations were small (≤ 0.33) or moderate (0.41 between the heating system and the type of the house, and 0.60 between the yard and the type of the house). The proportion of owner reported maternal history of atopic/allergic skin symptoms was 13.4% in the case group and 2.6% in the control group (S1 Table).

**Table 3:** Associations between potential risk factors (21 factors analysed) and owner-reported allergic/atopic skin symptoms in the DOGRISK questionnaire data.

	Potential risk factor	Included dogs (n)	Missing dogs (n)	Comparison categories	P-value	OR	95% CI	MV model
**1.**	Season of birth (reference / compared to: autumn)	8372	271	Winter	0.231	0.91	0.77–1.07	Included
				Spring	0.145*	0.89	0.76–1.04	
				Summer	0.147*	0.88	0.75–1.04	
**2.**	a) Heating system (reference: central heating)	7808	835	Wood-fired heating	**0.009**	**0.82**	0.70–0.95	Excluded; resumed in 2b (below)
				Heating with oil	0.362	0.91	0.75–1.11	
				Heating with ground heat	0.327	0.86	0.64–1.16	
	b) Wood-fired heating system (reference: no)	7808	835	Yes	**0.016**	**0.83**	0.72–0.97	Included
**3.**	Type of house the dog has previously lived in (reference: apartment)	6371	2272	Row house	0.240	0.90	0.76–1.07	Excluded: too many missing cases
				Detached house (wood)	**<0.001**	**0.72**	0.62–0.84	
				Detached house (not wood)	**0.001**	**0.70**	0.57–0.87	
**4.**	Type of house at the moment (reference: apartment)	8592	51	Row house	0.095*	0.88	0.76–1.02	Included
				Detached house (wood)	**<0.001**	**0.76**	0.66–0.87	
				Detached house (not wood)	**0.010**	**0.78**	0.64–0.94	
**5.**	a) Tidiness of the household (reference: extremely clean)	8592	51	Very clean	0.099*	0.72	0.48–1.07	Excluded: resumed in 5b (below)
				Normally clean	**0.025**	**0.66**	0.46–0.95	
				Not that clean	**0.015**	**0.62**	0.42–0.91	
				Not clean at all	0.891	0.96	0.51–1.81	
	b) Extremely clean household (reference: all others)	8592	51	Yes	**0.025**	**1.51**	1.05–2.15	Included
**6.**	Deworming status as a puppy (reference: yes)	7999	644	No	0.913	0.97	0.55–1.70	Excluded: non-significant
**7.**	Vaccination status as a puppy (reference: yes)	8223	420	No	0.471	1.22	0.71–2.08	Excluded: non-significant
**8.**	Dam’s deworming status pre-birth (reference: yes)	4061	4582	No	0.294	1.24	0.83–1.87	Excluded: non-significant and too many missing cases
**9.**	Dam’s vaccination status pre-birth (reference: yes)	2401	6242	No	0.901	1.02	0.80–1.28	Excluded: non-significant and too many missing cases
**10.**	Gender (reference: female)	8414	229	Male	0.119*	1.09	0.98–1.22	Included
**11.**	a) Colour of the coat (reference: very little/not at all white)	8188	455	90–100% white	**0.028**	**1.24**	1.02–1.50	Excluded; resumed in 11b (below)
				50–89% white	**0.012**	**1.25**	1.05–1.48	
				Less white	0.176*	0.91	0.80–1.04	
	b) Over 50% of white colour in the coat (reference: no)	8188	455	Yes	**<0.001**	**1.28**	1.13–1.47	Included
**12.**	Born in owner family (reference: no)	8643	0	Yes	**<0.001**	**0.33**	0.24–0.47	Included
**13.**	Living with other dogs (reference: no)	8643	0	Yes	**<0.001**	**0.71**	0.64–0.79	Included
**14.**	Living with other animals (reference: no)	8643	0	Yes	**0.028**	**0.88**	0.78–0.99	Included
**15.**	Where have you been smoking previously (reference: only outside)	3045	5598	Mainly inside	0.120*	0.62	0.33–1.14	Excluded: too many missing cases
				Rarely inside	0.408	1.16	0.82–1.66	
**16.**	Does the dog have a yard (reference: no)	8149	494	Yes a yard where the dog can be loose	0.051*	0.88	0.78–1.00	Included
				Yes an outside kennel where the dog can be loose	**<0.001**	**0.67**	0.55–0.81	
				Yes a yard where the dog is chained	0.169*	0.83	0.63–1.09	
**17.**	Body condition score under 2 months of age (reference: normal)	5846	2797	Obese	0.395	1.27	0.73–2.22	Excluded: too many missing cases
				Fat	**0.017**	**1.26**	1.04–1.52	
				Slim	0.963	1.01	0.79–1.28	
				Very slim	**0.022**	**1.84**	1.09–3.09	
**18.**	Outside under 2 months of age (reference: not at all)	5250	3393	Few days a month	0.605	1.09	0.78–1.54	Excluded: too many missing cases
				Few days a week	**0.018**	**0.70**	0.52–0.94	
				Once a day	**0.016**	**0.71**	0.53–0.94	
				Several times a day	**<0.001**	**0.61**	0.48–0.78	
**19.**	Walking outside when 5 months old (reference: under 30 min)	5839	2804	30–60 min/day	0.969	1.01	0.73–1.39	Excluded: too many missing cases
				1–2 hours/day	0.263	0.83	0.60–1.15	
				Over 2 hours per day	**0.012**	**0.63**	0.44–0.90	
**20.**	Dam having a history of skin symptoms (reference: no)	2944	5699	Yes	**<0.001**	**5.86**	4.03–8.51	Excluded: too many missing cases
**21.**	Age	8523	120		**<0.001**	**1.05**	1.03–1.07	Included

Results are based on univariable logistic regression analyses.

Missing dogs = the number of empty answers in the DOGRISK data; OR, odds ratio; CI, confidence interval; MV, multivariable; *, P < 0.2; **bolded**, P < 0.05.

Ten categorical risk factor candidates that presented P < 0.2 and less than 1500 missing cases were included in the multivariable logistic regression analysis (Table 4). These were the season of birth, the use of a wood-fired heating system, the type of house the dog was living in at the time, whether the household was extremely clean, gender, over 50% white colour in the coat, if the dog was born in owner family, if the dog is living with other dogs, if the dog is living with other animals, and if the dog has an outside kennel/yard. In addition, age and FCI breed groups (including mixed-breed dogs as 11^th^ group) were included in the model as adjusting variables. In the final model, the conditions ‘living with other dogs’, ‘born in the owner family’ and ‘living in a wooden or non-wooden detached house’ were associated with less owner-reported allergic/atopic skin symptoms, indicating that these traits were possibly protective. On the other hand, having an extremely clean household and over 50% white colour in the coat appeared to be positively associated with owner-reported allergic/atopic skin symptoms (Table 4). The model fit was verified by an Omnibus Chi-square of P <0.001 and a Nagelkerke’s R^2^ of 0.048. The Hosmer & Lemeshow test was 0.525. Overall prediction success was 81.6%.

**Table 4:** Associations of background variables, selected on the basis of univariable analyses, with owner-reported allergic/atopic skin symptoms in the DOGRISK questionnaire data (n = 5619).

	Risk factor	Comparison categories	P-value	OR	95% CI
**1.**	Living with other dogs (reference: no)	Yes	**<0.001**	**0.736**	0.642–0.844
**2.**	Born in owner family (reference: no)	Yes	**<0.001**	**0.369**	0.248–0.550
**3.**	Type of house at the moment (reference: apartment)	Wooden detached house	**0.009**	**0.699**	0.534–0.913
		Non-wooden detached house	**0.011**	**0.672**	0.495–0.913
**4.**	Over 50% white colour in the coat (reference: no)	Yes, over 50% of white colour	**0.019**	**1.216**	1.033–1.432
**5.**	Extremely clean household (reference: all others)	Yes	**0.032**	**1.596**	1.040–2.448

Results are based on a multiple logistic regression model adjusted for age and FCI breed group.

OR, odds ratio; CI, confidence interval.

The mean age of dogs with veterinary-verified CAD was 4.6 years (SD± 2.96). In the univariable analyses, using veterinary-verified CAD as the dependent variable, 6 of the 22 variables showed a statistically significant association with CAD. Factors that showed a protective association with CAD were: the type of house the dog had previously been living in (wooden detached house, P = 0.016; non-wooden detached house, P = 0.049), if the dog was born in the owner family (P = 0.003), and if the dog was living with other dogs (P = 0.001). On the other hand, over 50% white colour in the coat (P = 0.013), age (P = 0.001) and a dam having a history of allergic/atopic skin symptoms (P<0.001) were positively associated with veterinary-verified CAD.

Four categorical risk factor candidates that had P < 0.2 and less than 1500 missing cases were included in the multivariable logistic regression analysis: i) over 50% white colour in the coat; ii) if the dog was born in owner family; iii) if the dog was living with other dogs and iv) if the dog had an outside kennel/yard. In addition, age and FCI breed groups (including mixed-breed dogs as the 11^th^ group) were included in the model as adjusting variables. Altogether, 6407 dogs were included in the final model, including 6131 healthy dogs and 276 dogs with veterinary-verified CAD. In the final model living with other dogs (P = 0.002, OR = 0.665, 95% CI = 0.513–0.860) and being born in the owner family (P = 0.022), OR = 0.408, 95% CI = 0.189–0.880) showed a protective association with the development of CAD. The logistic regression showed an Omnibus Chi-square of P <0.001 and a Nagelkerke’s R^2^ of 0.043. The Hosmer & Lemeshow test was 0.138. The overall prediction success was 95.7%.

## Discussion

The results of the present study indicate that environmental factors related to household conditions (i.e. type of house, cleanliness of the household, other dogs in the household and being born in the owner family) and the colour of the dogs’ coat are associated with the owner-reported allergic/atopic skin symptoms. When these factors were considered among the subgroup of cases with veterinary-verified CAD two of them (if the dog was born in owner family and if it was living with other dogs) remained significant in the final model adjusted for other variables. At least three studies have analysed environmental factors related to CAD. The first studied insured dogs in Sweden, where the researchers had access to a large sample population but the diagnosis of the dogs was not necessarily accurate [39]. In the second study, where only 119 dogs of three different breeds were used, both the breeders and owners completed a questionnaire concerning dog nutrition and environment [40]. The third study included only Labrador and golden retrievers from Germany and Switzerland [23]. They had an overall of 378 dogs that were examined by a veterinary dermatologist, and the owners completed a 46-item questionnaire. Our study population of owner-reported allergic/atopic dogs included several thousands of individuals of different breeds covering all areas of Finland, but as in Nødtvedt et al. [39], not all dogs were diagnosed by a veterinarian. Hence, we also repeated the analyses using only dogs with veterinary-verified CAD as the test group.

According to previous literature, the breeds most commonly affected by CAD are West Highland white terriers, Labrador retrievers, golden retrievers, boxers, French bulldogs, German shepherds and cocker spaniels [4]. All of these breeds are also in the top 20 in our data suffering from owner-reported allergic/atopic skin symptoms, except for golden retriever being the 21^th^ and cocker spaniel as 27^th^. The reports of breed-associated diseases are often anecdotal, based on data from insurance companies or from referral centres, which makes them subject to sampling bias [41]. We had the same problem in our data: during recruitment, we contacted over 40 breed associations per year at dog shows and asked them to try to mobilize their members to answer the questionnaire and as the most active breed groups would had completed more questionnaires than others there may be a bias towards certain breeds. However, this bias should not have an impact on the percentage of owner-reported allergic/atopic skin symptoms within the breed, as both the owners of healthy and symptomatic dogs have answered the questionnaire. Additionally, since we only included breeds with at least 40 individuals in our data, rarer breeds with atopic predispositions may not have been included in the list.

The fact that some of the dogs were recruited from animal clinics giving them a leaflet in a waiting room may lead to a reporting or selection bias. The owner of a dog with an incident disease may remember and report the dog’s history in more detail or differently than an owner of an apparently healthy dog, and the owners visiting the selected clinics participating in the recruitment may differ from the average Finnish dog owners concerning the features of their living environment etc. Furthermore, the number of dogs with varying diseases in the control group may have been disproportionately large, and the distributions of risk factor variables for those diseases may differ from the overall dog population, again introducing a risk of bias. However, the majority of recruitment was conducted in a non-clinical setting.

When FCI breed groups were compared with mixed breed dogs, we found that groups 3 (Terriers) and 6 (Scent hounds and related breeds) had a significantly higher risk of owner-reported allergic/atopic skin symptoms than did mixed breed dogs; whereas the risk for groups 5 (Spitz and primitive types) and 10 (Sighthounds) were significantly lower (Table 3). The ten FCI breed groups are based on morphological and behavioural similarity, and historical function [42]. When classification is made based on genetic variation, four clusters have been reported [43]. The first cluster includes dogs from FCI groups 5 and 10, i.e. the groups with smaller risk of owner-reported allergic/atopic skin symptoms in our study. Their ancestry can be traced to both Asia and Africa, and they are also genetically closest to wolves [43]. The German shepherd (FCI 1) in cluster 4, Rhodesian ridgeback, beagle and bloodhound (FCI 6) in cluster 3, as well as some terriers (FCI 3) where the most genetically distant breeds from wolves [43]. These breeds and FCI groups all had a high proportion of owner-reported allergic/atopic skin symptoms in our study. This calls for further studies which would map candidate genes for canine skin diseases.

In this present study, living with other dogs was associated with a decreased risk of both veterinary-verified CAD and owner-reported allergic/atopic skin symptoms, which is in line with the study of CAD done by Meury et al. [23]. The hygiene hypothesis was introduced over 20 years ago, based on data showing that the incidence of eczema and hay fever in humans was lower in big families with a larger number of siblings [44] or brothers [45]. Similarly, the attendance to day care in the first year of life has been shown to decrease the risk of allergic diseases in children [46, 47]. It is hypothesized that the increased contact with other children at home or at day care increases the burden of infection, which has been reported to be inversely associated to atopy in humans [48–50]. Furthermore, having dogs or cats in the household during early childhood has been shown to protect against atopy in humans [5, 16–22], probably due to the increased exposure to microorganisms from soil and vegetation carried indoors by the dog. Nevertheless, conflicting results also exist as cats and dogs have even been reported to increase the risk of atopy in humans [21, 51–53]. Unfortunately, we have no data on whether the other dog(s) came to the household before or after the index dog, which makes it difficult to know if the index dog grew up with other dogs or if the other dogs came to the household later.

The multivariable regression model showed a significant association between the decreased risk both of veterinary-verified CAD and owner-reported allergic/atopic skin symptoms and being born in the owner family. This implies that there was at least one dog (the dam of the index dog) before in the family. This association could possibly be due to the fact that dog’s immune system adapts, from birth, to the microbial environment in which it is going to live for the rest of its life. When the dog is exposed to the microbes in the environment which affect the immune system already at a young age, it may thus decrease the prevalence of CAD later in life. This is also seen in human AD [50, 54]. Another explanation could be that stress affects skin symptoms, and that the presence of another dog may decrease the dog’s stress over time [55].

An extremely clean household was associated with a higher risk of owner-reported allergic/atopic skin symptoms when compared to a normally clean/unclean house in the present study. This also supports the hygiene hypothesis as constant cleaning reduces the microbial load and the level of endotoxins in the house, which has been reported to be inversely associated with allergic diseases in humans [56]. It should also be noted that the cleanliness of the household is subjective, and the term ‘extremely clean’ may differ between people. Hence, our result does not provide any specific frequency or intensity of cleaning that may have a positive association with atopic/allergic skin symptoms.

Living in a detached house compared to an apartment was also significantly associated with a lower risk of developing owner reported allergic/atopic skin symptoms in the final multivariable model. We also observed the similar results in our univariable regression analysis with the question ‘Type of house the dog has previously lived in’, with both owner-reported skin symptoms and veterinary-verified CAD. If allowing the amount of missing answers to be 2500 per question, the ‘Type of house the dog has previously lived in’ could be included in the multivariable model, and having a wooden detached house would be significantly associated with a decreased risk of owner-reported allergic/atopic skin symptoms and veterinary-verified CAD (data not shown). These findings are consistent with some human studies [9, 57]. People who live in detached houses also tend to live in suburban or rural areas. In a Swedish dog population, the occurrence of CAD among dogs living in urban areas was shown to be 57% higher than among the rest of the population [39], and the same research group reported an association between an increased human population density and a higher incidence rate of CAD [40]. A study among Labrador retrievers and golden retrievers also showed an association between living in a rural environment and decreased risk of AD [23]. There can be multiple reasons for the associations between rural environment and lower incidence of CAD, e.g. less exposure to diesel exhaust particles, better indoor air quality, more microbial contact, and more time spent outside. Also, more difficult access to specialist veterinary services outside city centres might affect the probability of a veterinary-verified CAD diagnosis.

Having over 50% white colour in the coat was associated with an increased risk of owner-reported allergic/atopic skin symptoms in the final multivariable model. When CAD was considered as the dependent variable, having over 50% white colour in the coat seemed to have a protective role in the univariable model but not in the final model. In the study of Nødtvedt et al. [40] a significantly higher incidence of AD was found in white bull terriers than those of other colours. No clear explanation for this association has been proposed so far. Recently, an association between c-*KIT*, a gene that partakes in mast cell development, and white spots on German shepherd dogs was found [58]. Strong connections between *c-KIT* and CAD in dogs [59, 60], as well as psoriasis [61] and asthma in humans [62] has also been reported. White Akita-inu dogs [63] and yellow Labrador and golden retrievers [64, 65] have been reported to be homozygous for the R306ter mutation of *MC1R*, a gene found in melanocytes. *MC1R* has also been reported to inhibit inflammation in atopic mice [66, 67]. In addition, canine β-defensin 103 (*cBD103*), a gene that is expressed by certain phagocytic leukocytes and epithelial cells [68], is found to be expressed in higher levels in black dog skin, and a mutation in the *cBD103* gene causes a black coat [69]. One study has reported a lower expression of cBD103 in both lesional and non-lesional skin of atopic dogs compared to healthy skin [70] but another study found no significant difference between atopic and healthy dog skin [68]. In the skin of human patients with AD, the expression of the human ortholog for cBD103, hBD3, is decreased [71–73]. Considering these results there might be a connection between coat colour and CAD, and anecdotal evidence supports it, but further research in this area is needed. Our result of coat colour and veterinary diagnosed CAD in a final model might also be due to chance or confounding factors.

The lack of a significant association between the season of birth with both veterinary-verified CAD and owner-reported allergic/atopic skin symptoms is consistent with a previous study of CAD [40], but the same research group also reported a significantly higher risk of CAD in autumn-born dogs [39]. Again, if allowing the amount of missing answers to be 2500 per question, then dogs born in summer were significantly less likely to have owner-reported allergic/atopic skin symptoms than dogs born in autumn (data not shown). No effect of gender on the risk of owner-reported allergic/atopic skin symptoms or verified CAD was detected in the present study. The most recent review agrees that CAD, in general, fails to exhibit sex predilection [74].

A history of owner-reported allergic/atopic skin symptoms in the dog’s dam was also a clear risk factor in our univariable regression analysis for both veterinary-verified CAD (P < 0.001; OR 4.35, CI 95% 2.16–8.76, data not shown), and owner-reported allergic/atopic skin symptoms even though the number of missing cases were high. Some dog breeds and breed groups are also more susceptible to atopy/allergies and skin symptoms than others, as can also be seen in different human ethnicities [75, 76]. It should also be considered that it is common for the dam to be born and to live in the breeder’s household as does the puppy for the first seven to eight weeks of its life. This way the dam and puppy share the same environmental conditions and nutrition, including in the gestation period. This introduces the possibility of mutual environmental factors affecting both the dam and the puppy in the womb as well as early in life [77, 78], in addition to shared genes.

There were some limitations to this study. When the pruritus is evaluated by the owners, it is always affected by their preconceived perception of what normal pruritic behaviour is. This can lead to an underestimation of skin problems and the owners to consider their dogs to be healthy. On the other hand, the dogs diagnosed as healthy by the veterinarian can be assessed by the owner as having pruritus above the ‘apparently normal’ level [79]. There are actions that correlate with higher pruritus, like paw licking/chewing, facial/muzzle rubbing, head shaking, and sneezing, but the owner may not associate this with pruritus and skin symptoms [79]. Also, some dogs may have clearly visible erythema but no signs of pruritus, or vice versa [80]. Considering the above, there is a substantial possibility that some of the control dogs in this study might have had the same conditions as the case dogs, like atopic dermatitis, and that their symptoms were not recognized by the owner. On the other hand, some case dogs that had owner-assessed pruritus might be considered healthy by a veterinarian. In addition, the owner-reported skin symptoms in the present study might have been due to parasite infestations, primary bacterial infections, stress, or something else unrelated to atopy/allergies, although the owners where specifically asked if they thought their dogs suffered from allergic/atopic skin symptoms. Parasite infestations that lead to scratching are, however, very rare in Finland.

Even though we had individuals with veterinary-verified CAD as a second population in this study, it is commonly known that both owners and veterinarians talk about the typical dermatological signs of atopy/allergy as CAD, even if the whole diagnostic procedure required had not been performed. Alternatively, a dog that fails to fulfil the criteria of atopic dermatitis in a clinical examination by the veterinarian may still be atopic [33]. Some of the associations between owner-reported allergic/atopic skin symptoms and risk factors were also found in the analyses of the veterinary-verified CAD test group (n = 322). The type of house the dog was living in at the moment, whether the household is extremely clean, and the colour of the dogs coat where not statistically significant in this test group, although they still may be associated with skin symptoms caused by CAD, since the number of cases were significantly smaller in these analyses.

Because this study was observational, the associations found between environmental factors and CAD / owner-reported allergic/atopic skin symptoms cannot be confirmed as causal. However, we found many statistically significant associations, most of which were consistent with previous human research and with previous studies in dogs suffering from CAD. To enrichen our findings, there will be dietary and genetic analyses performed within this DOGRISK study population. A follow-up questionnaire to the same Finnish dog population is under way and will shed better light on this issue, as it will enable a longitudinal approach.

## Conclusion

Based on the results of this study we conclude that living with other dogs, being born in the owner family, living in a detached house, and not having a predominantly white coat or living in an extremely clean household are associated with lower risk of owner-reported allergic/atopic skin symptoms, and possibly with veterinary-verified CAD. The owner-reported skin symptoms are most likely caused by atopic dermatitis and allergies, even if many of the dogs in the larger population lacked an official veterinary diagnosis. To our knowledge, our findings of an extremely clean household being associated with a higher risk of skin symptoms is novel in dogs. We also report new information regarding the association of different FCI breed groups and skin symptoms. This shows that genetic factors also contribute to the predisposition for skin problems. An important question for future studies is the relative contribution of genetics, dietary factors and other environmental factors have on the risk of canine and human AD.

## Supporting information

S1 Table Distributions of all the categorical variables including their categories used in the analyses for dogs with owner-reported allergic/atopic skin symptoms (cases, n = 1585), dogs without owner-reported allergic/atopic skin symptoms (controls, n = 7058) and all dogs from the DOGRISK questionnaire. (DOCX)

## References

[1] Ka D, Marignac G, Desquilbet L, Freyburger L, Hubert B, Garelik D, et al Association between passive smoking and atopic dermatitis in dogs. Food Chem Toxicol. 2014;66: 329–333. 10.1016/j.fct.2014.01.015 24491262

[2] Halliwell R. Revised nomenclature for veterinary allergy. Vet. Immunol. Immunopathol. 2006;114: 207–208. 10.1016/j.vetimm.2006.08.013 17005257

[3] Jordaan HF, Todd G, Sinclair W, Green RJ. Aetiopathogenesis of atopic dermatitis. S Afr Med J. 2014;104: 706–709. 25538994 10.7196/samj.8840

[4] Bizikova P, Pucheu-Haston C, Eisenschenk MNC, Marsella R, Nuttall T, Santoro D. Review: Role of genetics and the environment in the pathogenesis of canine atopic dermatitis. Vet Dermatol. 2015;26: 95–e26. 10.1111/vde.12198 25703290

[5] Biagini Myers JM, Wang N, Lemasters GK, Bernstein DI, Epstein TG, Lindsey MA, et al Genetic and environmental risk factors for childhood eczema development and allergic sensitization in the CCAAPS cohort. J Invest Dermatol. 2010;130: 430–437. 10.1038/jid.2009.300 19759553 PMC2807898

[6] Cooper PJ, Chico ME, Rodrigues LC, Ordonez M, Strachan D, Griffin GE, et al Reduced risk of atopy among school-age children infected with geohelminth parasites in a rural area of the tropics. J Allergy Clin Immunol. 2003;111: 995–1000. 12743563 10.1067/mai.2003.1348

[7] Yemaneberhan H, Flohr C, Lewis SA, Bekele Z, Parry E, Williams HC, et al Prevalence and associated factors of atopic dermatitis symptoms in rural and urban Ethiopia. Clinical & Experimental Allergy 2004;34: 779–785. 15144471 10.1111/j.1365-2222.2004.1946.x

[8] Flohr C, Pascoe D, Williams HC. Atopic dermatitis and the ‘hygiene hypothesis’: Too clean to be true? Br J Dermatol. 2005;152: 202–216. 10.1111/j.1365-2133.2004.06436.x 15727630

[9] Zeyrek CD, Zeyrek F, Sevinc E, Demir E. Prevalence of asthma and allergic diseases in Sanliurfa, Turkey, and the relation to environmental and socioeconomic factors: Is the hygiene hypothesis enough? J Investig Allergol Clin Immunol. 2006;16: 290–295. 17039667

[10] Schram ME, Tedja AM, Spijker R, Bos JD, Williams HC, Spuls PI. Is there a rural/urban gradient in the prevalence of eczema? A systematic review. Br J Dermatol. 2010;162: 964–973. 10.1111/j.1365-2133.2010.09689.x 20331459

[11] Xu F, Yan S, Li F, Cai M, Chai W, Wu M, et al Prevalence of childhood atopic dermatitis: An urban and rural community-based study in shanghai, china. PLoS One. 2012;7: e36174 10.1371/journal.pone.0036174 22563481 PMC3341360

[12] Lampi J, Canoy D, Jarvis D, Hartikainen A, Keski-Nisula L, Järvelin M, et al Farming environment and prevalence of atopy at age 31: Prospective birth cohort study in Finland. Clin Exp Allergy. 2011;41: 987–993. 10.1111/j.1365-2222.2011.03777.x 21575087

[13] Ege MJ, Mayer M, Normand A, Genuneit J, Cookson WOCM, Braun-Fahrländer C, et al Exposure to environmental microorganisms and childhood asthma. N Engl J Med. 2011;364: 701–709. 10.1056/NEJMoa1007302 21345099

[14] Illi S, Depner M, Genuneit J, Horak E, Loss G, Strunz-Lehner C, et al Protection from childhood asthma and allergy in alpine farm environments-the GABRIEL advanced studies. J Allergy Clin Immunol. 2012;129: 1470–1477.e6. 10.1016/j.jaci.2012.03.013 22534534

[15] Horak E, Morass B, Ulmer H, Genuneit J, Braun-Fahrlander C, von Mutius E. Prevalence of wheezing and atopic diseases in Austrian schoolchildren in conjunction with urban, rural or farm residence. Wien Klin Wochenschr. 2014;126: 532–536. 10.1007/s00508-014-0571-z 25047409

[16] Johnson CC, Alford SH. Do animals on the farm and in the home reduce the risk of pediatric atopy? Curr Opin Allergy Clin Immunol. 2002;2: 133–139. 11964762 10.1097/00130832-200204000-00009

[17] Bisgaard H, Halkjaer LB, Hinge R, Giwercman C, Palmer C, Silveira L, et al Risk analysis of early childhood eczema. J Allergy Clin Immunol. 2009;123: 1355–1360.e5. 10.1016/j.jaci.2009.03.046 19501236

[18] Almqvist C, Garden F, Kemp AS, Li Q, Crisafulli D, Tovey ER, et al Effects of early cat or dog ownership on sensitisation and asthma in a high-risk cohort without disease-related modification of exposure. Paediatr Perinat Epidemiol. 2010;24: 171–178. 10.1111/j.1365-3016.2010.01095.x 20415774

[19] Pelucchi C, Galeone C, Bach JF, La Vecchia C, Chatenoud L. Pet exposure and risk of atopic dermatitis at the pediatric age: A meta-analysis of birth cohort studies. J Allergy Clin Immunol. 2013;132: 616–622.e7. 10.1016/j.jaci.2013.04.009 23711545

[20] Carson CG. Risk factors for developing atopic dermatitis. Dan Med J. 2013;60: B4687 23809981

[21] Takkouche B, González-Barcala F, Etminan M, FitzGerald M. Exposure to furry pets and the risk of asthma and allergic rhinitis: A meta-analysis. Allergy. 2008;63: 857–864. 10.1111/j.1398-9995.2008.01732.x 18588551

[22] Roduit C, Wohlgensinger J, Frei R, Bitter S, Bieli C, Loeliger S, et al Prenatal animal contact and gene expression of innate immunity receptors at birth are associated with atopic dermatitis. J. Allergy Clin. Immunol. 2011;127: 179–185, 185.e1 10.1016/j.jaci.2010.10.010 21112617

[23] Meury S, Molitor V, Doherr MG, Roosje P, Leeb T, Hobi S, et al Role of the environment in the development of canine atopic dermatitis in Labrador and golden retrievers. Vet Dermatol. 2011;22: 327–334. 10.1111/j.1365-3164.2010.00950.x 21251098

[24] Tamsmark TH, Koch A, Melbye M, Mùlbak K. Incidence and predictors of atopic dermatitis in an open birth cohort in Sisimiut, Greenland. Acta Paediatrica 2001;90: 982–988. 11683210 10.1111/j.1651-2227.2001.tb01352.x

[25] Purvis DJ, Thompson JMD, Clark PM, Robinson E, Black PN, Wild CJ, et al Risk factors for atopic dermatitis in New Zealand children at 3·5 years of age. Br J Dermatol. 2005;152: 742–749. 10.1111/j.1365-2133.2005.06540.x 15840107

[26] Morar N, Willis-Owen SA, Moffatt MF, Cookson WO. The genetics of atopic dermatitis. J Allergy Clin Immunol. 2006;118: 24–34. 10.1016/j.jaci.2006.03.037 16815134

[27] von Hertzen L, Makela MJ, Petays T, Jousilahti P, Kosunen TU, Laatikainen T, et al Growing disparities in atopy between the Finns and the Russians: A comparison of 2 generations. J Allergy Clin Immunol. 2006;117: 151–157. 10.1016/j.jaci.2005.07.028 16387599

[28] Shams K, Grindlay DJC, Williams HC. What's new in atopic eczema? An analysis of systematic reviews published in 2009–2010. Clin Exp Dermatol. 2011;36: 573–578. 10.1111/j.1365-2230.2011.04078.x 21718344

[29] DeBoer DJ, Marsella R. The ACVD task force on canine atopic dermatitis (XII): The relationship of cutaneous infections to the pathogenesis and clinical course of canine atopic dermatitis. Vet Immunol Immunopathol. 2001;81: 239–249. 11553386 10.1016/s0165-2427(01)00345-2

[30] Fazakerley J, Nuttall T, Sales D, Schmidt V, Carter SD, Hart CA, et al Staphylococcal colonization of mucosal and lesional skin sites in atopic and healthy dogs. Vet Dermatol. 2009;20: 179–184. 10.1111/j.1365-3164.2009.00745.x 19392768

[31] Wynchank D, Berk M. Fluoxetine treatment of acral lick dermatitis in dogs: A placebo-controlled randomized double blind trial this paper was presented at the XX CINP congress, Melbourne, June 23–27, 1996. Depression & Anxiety (1091–4269) 1998;8: 21–23. 9750975

[32] Bond R, Ferguson EA, Curtis CF, Craig JM. Lloyd DH. Factors associated with elevated cutaneous *Malassezia pachydermatis* populations in dogs with pruritic skin disease. J Small Anim Pract. 1996;37: 103–107. 8683952 10.1111/j.1748-5827.1996.tb02353.x

[33] Nødtvedt A, Bergvall K, Emanuelson U, Egenvall A. Canine atopic dermatitis: Validation of recorded diagnosis against practice records in 335 insured Swedish dogs. Acta Vet Scand. 2006;47: 8–7. 10.1186/1751-0147-48-8 PMC1553461 16987404

[34] Saari S, Näreaho A, Nikander S. Elinympäristönä koira—koiran loiset ja loissairaudet, First Ed., Fennovet OY, Helsinki; 2016 (Textbook only in Finnish).

[35] Scott DW, Miller WH, Griffin CE. (Eds.). Muller and Kirk’s Small Animal Dermatology. 6th Ed WB Saunders Co, Philadelphia, Pa; 2001 pp 457–473.

[36] Roine J, Uusitalo L, Hielm-Björkman A. Validating and reliability testing the descriptive data and three different disease diagnoses of the internet-based DOGRISK questionnaire. BMC Vet Res. 2016;12: 30 10.1186/s12917-016-0658-z 26897627 PMC4761135

[37] FCI, 2010. FCI Fédération Cynologique Internationale. Standards and nomenclature. 2010. http://www.fci.be/en/nomenclature/. Accessed 14 November, 2014.

[38] Dohoo I, Martin W, Stryhn H. Methods in epidemiologic research. VER Inc Charlottetown, Canada 2012 pp 413, 499–500.

[39] Nødtvedt A, Egenvall A, Bergvall K, Hedhammar Å. Incidence of and risk factors for atopic dermatitis in a Swedish population of insured dogs. Veterinary Record: Journal of the British Veterinary Association 2006;159: 241–246. 10.1136/vr.159.8.241 16921013

[40] Nødtvedt A, Bergvall K, Sallander M, Egenvall A, Emanuelson U, Hedhammar Å. A case—control study of risk factors for canine atopic dermatitis among boxer, bullterrier and West Highland white terrier dogs in Sweden. Vet Dermatol. 2007;18: 309–315. 10.1111/j.1365-3164.2007.00617.x 17845618

[41] Clements DN, Handel IG, Rose E, Querry D, Pugh CA, Ollier WER, et al Dogslife: A web-based longitudinal study of Labrador retriever health in the UK. BMC Vet Res. 2013;9: 1–15. 23332044 10.1186/1746-6148-9-13 PMC3559277

[42] Turcsán B, Kubinyi E, Miklósi Á. Trainability and boldness traits differ between dog breed clusters based on conventional breed categories and genetic relatedness. Appl Anim Behav Sci. 2011;132: 61–70.

[43] Parker HC, Kim LV, Suiter NB, Carlson S, Lorentzen TD, Malek TB, et al Genetic structure of the purebred domestic dog. Science. 2004;304: 1160–1164. 10.1126/science.1097406 15155949

[44] Strachan DP. Hay fever, hygiene, and household size. BMJ: British Medical Journal (International Edition) 1989;299: 1259. 10.1136/bmj.299.6710.1259 PMC1838109 2513902

[45] Svanes C, Jarvis D, Chinn S, Burney P. Childhood environment and adult atopy: results from the European Community Respiratory Health Survey. J Allergy Clin Immunol. 1999;103:415–420. 10069874 10.1016/s0091-6749(99)70465-3

[46] Celedon JC, Wright RJ, Litonjua AA, Sredl D, Ryan L, Weiss ST, et al Day care attendance in early life, maternal history of asthma, and asthma at the age of 6 years. Am J Respir Crit Care Med. 2003;167: 1239–1243. 10.1164/rccm.200209-1063OC 12446273

[47] Rothers J, Stern DA, Spangenberg A, Lohman IC, Halonen M, Wright AL. Influence of early day-care exposure on total IgE levels through age 3 years. J Allergy Clin Immunol. 2007;120: 1201–1207. 10.1016/j.jaci.2007.07.036 17854882

[48] Linneberg A, Ostergaard C, Tvede M, Andersen LP, Nielsen NH, Madsen F, et al IgG antibodies against microorganisms and atopic disease in Danish adults: The Copenhagen allergy study. J Allergy Clin Immunol. 2003;111: 847–853. 12704368 10.1067/mai.2003.1335

[49] von Hertzen LC, Laatikainen T, Mäkelä MJ, Jousilahti P, Kosunen TU, Petäys T, et al Infectious burden as a determinant of atopy—A comparison between adults in Finnish and Russian Karelia. Int Arch Allergy Immunol. 2006;140: 89–95. 10.1159/000092251 16554659

[50] Janson C, Asbjornsdottir H, Birgisdottir A, Sigurjonsdottir RB, Gunnbjornsdottir M, Gislason D, et al The effect of infectious burden on the prevalence of atopy and respiratory allergies in Iceland, Estonia, and Sweden. J Allergy Clin Immunol. 2007;120: 673–679. 10.1016/j.jaci.2007.05.003 17586034

[51] Ownby DR, Johnson CC, Peterson EL. Exposure to dogs and cats in the first year of life and risk of allergic sensitization at 6 to 7 years of age. JAMA. 2002;288: 963 12190366 10.1001/jama.288.8.963

[52] Bisgaard H, Simpson A, Palmer CNA, Bnnelykke K, Mclean I, Mukhopadhyay S, et al Gene-environment interaction in the onset of eczema in infancy: Filaggrin loss-of-function mutations enhanced by neonatal cat exposure. PLoS Med. 2008;5: e131 10.1371/journal.pmed.0050131 18578563 PMC2504043

[53] Epstein TG, Bernstein DI, Levin L, Khurana Hershey GK, Ryan PH, Reponen T, et al Opposing effects of cat and dog ownership and allergic sensitization on eczema in an atopic birth cohort. J Pediatr. 2011;158: 265–271.e1–5. 10.1016/j.jpeds.2010.07.026 20884006 PMC4910508

[54] Romagnani S. The increased prevalence of allergy and the hygiene hypothesis: Missing immune deviation, reduced immune suppression, or both? Immunology. 2004;112: 352–363. 10.1111/j.1365-2567.2004.01925.x 15196202 PMC1782506

[55] Beerda B, Schilder MB, van Hooff JA, de Vries HW, Mol JA. Chronic stress in dogs subjected to social and spatial restriction. I. Behavioral responses. Physiol Behav. 1999;66:233–242. 10336149 10.1016/s0031-9384(98)00289-3

[56] Tse K, Horner A. Defining a role for ambient TLR ligand exposures in the genesis and prevention of allergic diseases. Seminars in Immunopathology 2008;30: 53–62. 10.1007/s00281-007-0098-8 17989979

[57] Lee JH, Suh J, Kim EH, Cho JB, Park HY, Kim J, et al Surveillance of home environment in children with atopic dermatitis: A questionnaire survey. Asia Pac Allergy 2012;2: 59–66. 10.5415/apallergy.2012.2.1.59 22348208 PMC3269603

[58] Wong AK, Ruhe AL, Robertson KR, Loew ER, Williams DC, Neff MW. A de novo mutation in KIT causes white spotting in a subpopulation of German shepherd dogs. Anim Genet. 2013;44: 305–310. 10.1111/age.12006 23134432

[59] Daigle J, Moussy A, Mansfield CD, Hermine O. Masitinib for the treatment of canine atopic dermatitis: A pilot study. Vet Res Commun. 2010;34: 51–63. 10.1007/s11259-009-9332-2 20033487

[60] Cadot P, Hensel P, Bensignor E, Hadjaje C, Marignac G, Beco L, et al Masitinib decreases signs of canine atopic dermatitis: A multicentre, randomized, double-blind, placebo-controlled phase 3 trial. Vet Dermatol. 2011;22: 554–564. 10.1111/j.1365-3164.2011.00990.x 21668810

[61] Huttunen M, Naukkarinen A, Horsmanheimo M, Harvima IT. Transient production of stem cell factor in dermal cells but increasing expression of kit receptor in mast cells during normal wound healing. Arch Dermatol Res. 2002;294: 324–330. 12373338 10.1007/s00403-002-0331-1

[62] Al-Muhsen S, Shablovsky G, Olivenstein R, Mazer B, Hamid Q. The expression of stem cell factor and c-kit receptor in human asthmatic airways. Clin Exp Allergy. 2004;34: 911–916. 10.1111/j.1365-2222.2004.01975.x 15196279

[63] Oguro-Okano M, Honda M, Yamazaki K, Okano K. Mutations in the melanocortin 1 receptor, beta-defensin103 and agouti signaling protein genes, and their association with coat color phenotypes in akita-inu dogs. J Vet Med Sci. 2011;73: 853–858. 21321476 10.1292/jvms.10-0439

[64] Everts RE, Rothuizen J, Oost BA. Identification of a premature stop codon in the melanocyte-stimulating hormone receptor gene (MC1R) in Labrador and golden retrievers with yellow coat colour. Anim Genet. 2000;31: 194–199. 10895310 10.1046/j.1365-2052.2000.00639.x

[65] Newton JM, Cohen-Barak O, Hagiwara N, Gardner JM, Davisson MT, King RA, et al Mutations in the human orthologue of the mouse underwhite gene (uw) underlie a new form of oculocutaneous albinism, OCA4. Am J Hum Genet. 2001;69: 981–988. 10.1086/324340 11574907 PMC1274374

[66] Etori M, Yonekubo K, Sato E, Mizukami K, Hirahara K, Karasuyama H, et al Melanocortin receptors 1 and 5 might mediate inhibitory effects of α-melanocyte-stimulating hormone on antigen-induced chronic allergic skin inflammation in IgE transgenic mice. J Invest Dermatol. 2012;132: 1925–1927. 10.1038/jid.2012.68 22437312

[67] Chen W, Li J, Qu H, Song Z, Yang Z, Huo J, et al The melanocortin 1 receptor (MC1R) inhibits the inflammatory response in raw 264.7 cells and atopic dermatitis (AD) mouse model. Mol Biol Rep. 2013;40: 1987–1996. 10.1007/s11033-012-2256-x 23090482

[68] Leonard BC, Marks SL, Outerbridge CA, Affolter VK, Kananurak A, Young A, et al Activity, expression and genetic variation of canine beta-defensin 103: A multifunctional antimicrobial peptide in the skin of domestic dogs. J Innate Immun. 2012;4: 248–259. 10.1159/000334566 22261569 PMC3357142

[69] Candille SI, Kaelin CB, Cattanach BM, Vu B, Thompson DA, Nix MA, et al A ß-defensin mutation causes black coat color in domestic dogs. Science. 2007;318: 1418–1423. 10.1126/science.1147880 17947548 PMC2906624

[70] van Damme CMM, Willemse T, van Dijk A, Haagsman HP, Veldhuizen EJA. Altered cutaneous expression of β-defensins in dogs with atopic dermatitis. Mol Immunol. 2009;46: 2449–2455. 10.1016/j.molimm.2009.05.028 19576634

[71] Ong PY, Ohtake T, Brandt C, Wood RA. Endogenous antimicrobial peptides and skin infections in atopic dermatitis. Pediatrics. 2003;112: 461. 10.1056/NEJMoa021481 12374875

[72] Howell MD, Boguniewicz M, Pastore S, Novak N, Bieber T, Girolomoni G, Leung DYM. Mechanism of HBD-3 deficiency in atopic dermatitis. Clin Immunol. 2006;121: 332–338. 10.1016/j.clim.2006.08.008 17015038

[73] Kisich KO, Carspecken CW, Fieve S, Boguniewicz M, Leung DY. Defective killing of staphylococcus aureus in atopic dermatitis is associated with reduced mobilization of human beta-defensin-3. J Allergy Clin Immunol. 2008;122: 62–68. 10.1016/j.jaci.2008.04.022 18538383

[74] Bizikova P, Santoro D, Marsella R, Nuttall T, Eisenschenk MNC, Pucheu-Haston C. Review: Clinical and histological manifestations of canine atopic dermatitis. Vet Dermatol 2015;26: 79–e24. 10.1111/vde.12196 25676252

[75] Williams HC, Pembroke AC, Forsdyke H, Boodoo G, Hay RJ, Burney PG. London-born black Caribbean children are at increased risk of atopic dermatitis. J Am Acad Dermatol. 1995;32: 212–217. 7829705 10.1016/0190-9622(95)90128-0

[76] Torrelo A. Atopic dermatitis in different skin types. What is to know? Journal of the European Academy of Dermatology & Venereology 2014;28: 2–4. 24702443 10.1111/jdv.12480

[77] Muñoz M, Pong-Wong R, Canela-Xandri O, Rawlik K, Haley CS, Tenesa A. Evaluating the contribution of genetics and familial shared environment to common disease using the UK Biobank. Nat Genet. 2016;48:980–983. 10.1038/ng.3618 27428752 PMC5989924

[78] Martino DJ, Prescott SL. Progress in understanding the epigenetic basis for immune development, immune function, and the rising incidence of allergic disease. Curr Allergy Asthma Rep. 2013;13:85–92. 10.1007/s11882-012-0312-1 23054626

[79] Stetina KM, Marks SL, Griffin CE. Owner assessment of pruritus and gastrointestinal signs in apparently healthy dogs with no history of cutaneous or noncutaneous disease. Vet Dermatol. 2015;26: 246–e54. 10.1111/vde.12219 26178605

[80] Hill P, Rybníček J, Lau-Gillard P. Correlation between pruritus score and grossly visible erythema in dogs. Vet Dermatol. 2010;21: 450–455. 10.1111/j.1365-3164.2010.00881.x 20456720

