# Supplementary material for: Environmental and phenotype-related risk factors for owner-reported allergic/atopic skin symptoms and for canine atopic dermatitis verified by veterinarian in a Finnish dog population
Source: PLoS One. 2017 Jun 1;12(6):e0178771. doi: 10.1371/journal.pone.0178771 (PMC5453595; doi:10.1371/journal.pone.0178771)
Supplement: S1 Table — (DOCX) [file pone.0178771.s001.docx]

**S1 Table. Distributions of all the categorical variables including their categories used in the analyses for dogs with owner-reported allergic/atopic skin symptoms (cases, n=1585), dogs without owner-reported allergic/atopic skin symptoms (controls, n=7058) and all dogs from the DOGRISK questionnaire.**

|  | Tested risk factor | Categories | Cases % (n) | Controls % (n) | All dogs |
| --- | --- | --- | --- | --- | --- |
| 1. | Season of birth | Autumn | 23.1 (353) | 21.1 (1446) | 21.5 (1799) |
|  |  | Winter | 24.1 (369) | 24.4 (1669) | 24.3 (2038) |
|  |  | Spring | 29.8 (456) | 30.6 (2095) | 30.5 (2551) |
|  |  | Summer | 23.0 (351) | 23.9 (1633) | 23.7 (1984) |
| 2. | Wood-fired heating system | Yes | 17.4 (251) | 20.3 (1290) | 19.7 (1541) |
|  |  | No | 82.6 (1188) | 79.7 (5079) | 80.3 (6267) |
| 3. | Type of house the dog has previously lived in | Apartment | 39.9 (493) | 33.7 (1732) | 34.9 (2225) |
|  |  | Row house | 20.0 (247) | 18.7 (962) | 19.0 (1209) |
|  |  | Detached house (wood) | 29.7 (367) | 34.9 (1794) | 33.9 (2161) |
|  |  | Detached house (not wood) | 10.4 (129) | 12.6 (647) | 12.2 (776) |
| 4. | Type of house at the moment | Apartment | 35.3 (557) | 30.3 (2127) | 31.2 (2684) |
|  |  | Row house | 21.0 (331) | 20.5 (1438) | 20.6 (1769) |
|  |  | Detached house (wood) | 33.4 (527) | 37.8 (2654) | 37.0 (3181) |
|  |  | Detached house (not wood) | 10.3 (162) | 10.3 (162) | 11.1 (958) |
| 5. | Extremely clean household | Yes | 2.6 (41) | 1.7 (122) | 1.9 (163) |
|  |  | No | 97.4 (1538) | 98.3 (6891) | 98.1 (8429) |
| 6. | Deworming status as a puppy | Yes | 99.0 (1429) | 99.0 (6489) | 99.0 (7918) |
|  |  | No | 1.0 (15) | 1.0 (66) | 1.0 (81) |
| 7. | Vaccination status as a puppy | Yes | 98.9 (1472) | 98.7 (6647) | 98.7 (8119) |
|  |  | No | 1.1 (16) | 1.3 (88) | 1.3 (104) |
| 8. | Dam’s deworming status pre-birth | Yes | 95.4 (619) | 96.2 (3284) | 96.1 (3903) |
|  |  | No | 4.6 (30) | 3.8 (128) | 3.9 (158) |
| 9. | Dam’s vaccination status pre-birth | Yes | 51.4 (169) | 51.7 (1072) | 51.7 (1241) |
|  |  | No | 48.6 (160) | 48.3 (1000) | 48.3 (1160) |
| 10. | Gender | Male | 48.9 (754) | 46.7 (3207) | 47.1 (3961) |
|  |  | Female | 51.1 (789) | 53.3 (3664) | 52.9 (4453) |
| 11. | Over 50 % of white colour in the coat | Yes | 24.1 (364) | 19.8 (1321) | 20.6 (1685) |
|  |  | No | 75.9 (1149) | 80.2 (5354) | 79.4 (6503) |
| 12. | Born in owner family | Yes | 2.5 (39) | 7.0 (495) | 6.2 (534) |
|  |  | No | 97.5 (1546) | 93.0 (6563) | 93.8 (8109) |
| 13. | Living with other dogs | Yes | 39.8 (821) | 60.2 (4250) | 58.7 (5071) |
|  |  | No | 48.2 (764) | 30.1 (1827) | 41.3 (3572) |
| 14. | Living with other animals | Yes | 31.5 (500) | 34.4 (2430) | 33.9 (2930) |
|  |  | No | 68.5 (1085) | 65.6 (4628) | 66.1 (5713) |
| 15. | Where have you been smoking previously | Only outside | 90.8 (563) | 90.6 (2224) | 90.6 (2760) |
|  |  | Rarely inside | 7.1 (42) | 6.1 (150) | 6.3 (192) |
|  |  | Mainly inside | 2.0 (12) | 3.3 (81) | 3.1 (93) |
| 16. | Does the dog have a yard | Yes a yard where the dog can be loose | 49.5 (738) | 49.6 (3304) | 49.6 (4042) |
|  |  | Yes an outside kennel where the dog can be loose | 10.7 (159) | 14.2 (946) | 13.6 (1105) |
|  |  | Yes a yard where the dog is chained | 4.8 (71) | 5.1 (340) | 5.0 (411) |
|  |  | No | 35.1 (523) | 31.1 (2068) | 31.8 (2591) |
| 17. | Body condition score under 2 months of age | Obese | 1.5 (16) | 1.3 (60) | 1.3 (76) |
|  |  | Fat | 15.6 (164) | 13.0 (622) | 13.4 (786) |
|  |  | Normal | 72.7 (765) | 76.1 (3648) | 75.5 (4412) |
|  |  | Slim | 8.3 (87) | 8.6 (413) | 8.6 (500) |
|  |  | Very slim | 1.9 (20) | 1.1 (52) | 1.2 (72) |
| 18. | Outside under 2 months of age | Not at all | 11.8 (106) | 8.2 (357) | 8.8 (463) |
|  |  | Few days a month | 8.4 (75) | 5.3 (231) | 5.8 (306) |
|  |  | Few days a week | 12.6 (113) | 12.5 (544) | 12.5 (657) |
|  |  | Once a day | 15.1 (135) | 14.8 (645) | 14.9 (780) |
|  |  | Several times a day | 52.2 (468) | 59.2 (2576) | 58.0 (3044) |
| 19. | Walking outside when 5 months old | Under 30 min. | 4.8 (51) | 4.2 (198) | 4.3 (249) |
|  |  | 30-60 min/day | 41.9 (448) | 36.2 (1728) | 37.3 (2176) |
|  |  | 1-2 hours/day | 41.6 (444) | 43.5 (2075) | 43.1 (2519) |
|  |  | Over 2 hours per day | 11.7 (125) | 16.1 (770) | 15.3 (895) |
| 20. | Dam having a history of skin symptoms | Yes | 13.4 (56) | 2.6 (65) | 4.1 (121) |
|  |  | No | 86.6 (362) | 97.4 (2461) | 95.9 (2823) |
| 21. | FCI breed groups | 1 | 3.3 (279) | 15.5 (1317) | 18.8 (1596) |
|  |  | 2 | 3.1 (266) | 12.3 (1046) | 15.4 (1312) |
|  |  | 3 | 2.7 (227) | 7.8 (662) | 10.5 (889) |
|  |  | 4 | 0.3 (23) | 1.6 (136) | 1.9 (159) |
|  |  | 5 | 1.4 (119) | 9.4 (796) | 10.8 (915) |
|  |  | 6 | 0.6 (51) | 1.7 (145) | 2.3 (196) |
|  |  | 7 | 0.4 (30) | 1.8 (154) | 2.2 (184) |
|  |  | 8 | 2.3 (199) | 10.2 (871) | 12.6 (1070) |
|  |  | 9 | 1.7 (143) | 7.8 (662) | 9.5 (805) |
|  |  | 10 | 0.3 (22) | 2.3 (199) | 2.6 (221) |
|  | Mixed-breed dogs | 11 | 2.4 (200) | 11.2 (954) | 13.6 (1154) |

n, number of dogs; FCI breed groups: 1, Sheepdogs and Cattledogs (exp. Swiss Cattledogs); 2, Pinscher and Schnauzer – Molossoid and Swiss Mountain and Cattledogs; 3, Terriers; 4, Dachshunds; 5, Spitz and primitive types; 6, Scent hounds and related breeds; 7, Pointing Dogs; 8, Retrievers – Flushing Dogs – Water Dogs; 9, Companion and Toy Dogs; 10, Sighthounds.
